# Carbon Nanomaterials and LED Irradiation as Antibacterial Strategies against Gram-Positive Multidrug-Resistant Pathogens

**DOI:** 10.3390/ijms20143603

**Published:** 2019-07-23

**Authors:** Lisa Elias, Rafael Taengua, Belén Frígols, Beatriz Salesa, Ángel Serrano-Aroca

**Affiliations:** 1 Department of Biochemistry and Biotechnology, Ghent University, Krijgslaan 281, 9000 Ghent, Belgium; 2 Facultad de Veterinaria y Ciencias Experimentales, Universidad Católica de Valencia San Vicente Mártir, c/Guillem de Castro 94, 46001 Valencia, Spain

**Keywords:** carbon nanomaterials, antibacterial activity, graphene oxide, carbon nanofibers, LED, methicillin-resistant *Staphylococcus aureus*, methicillin-resistant *Staphylococcus epidermidis*, cytotoxicity, human keratinocyte HaCaT cells

## Abstract

Background: Due to current antibiotic resistance worldwide, there is an urgent need to find new alternative antibacterial approaches capable of dealing with multidrug-resistant pathogens. Most recent studies have demonstrated the antibacterial activity and non-cytotoxicity of carbon nanomaterials such as graphene oxide (GO) and carbon nanofibers (CNFs). On the other hand, light-emitting diodes (LEDs) have shown great potential in a wide range of biomedical applications. Methods: We investigated a nanotechnological strategy consisting of GO or CNFs combined with light-emitting diod (LED) irradiation as novel nanoweapons against two clinically relevant Gram-positive multidrug-resistant pathogens: methicillin-resistant *Staphylococcus aureus* (MRSA) and methicillin-resistant *Staphylococcus epidermidis* (MRSE). The cytotoxicity of GO and CNFs was studied in the presence of human keratinocyte HaCaT cells. Results: GO or CNFs exhibited no cytotoxicity and high antibacterial activity in direct contact with MRSE and MRSA cells. Furthermore, when GO or CNFs were illuminated with LED light, the MRSE and MRSA cells lost viability. The rate of decrease in colony forming units from 0 to 3 h, measured per mL, increased to 98.5 ± 1.6% and 95.8 ± 1.4% for GO and 99.5 ± 0.6% and 99.7 ± 0.2% for CNFs. Conclusions: This combined antimicrobial approach opens up many biomedical research opportunities and provides an enhanced strategy for the prevention and treatment of Gram-positive multidrug-resistant infections.

## 1. Introduction

Due to the excessive use of antibiotics, hospital-associated infections are more increasingly spreading without any suitable treatment [1]. In fact, the World Health Organization has recently announced the increasing high levels of antibiotic resistance (AR) to very important pathogens [2]. Furthermore, considering the rapidly growing rate of AR in various pathogens, it is estimated that this problem could surpass all other life-threatening diseases including cancer by 2050 [3] if no proactive solutions are found to slow down the rise of drug resistance or if no alternative antibacterial strategies are successfully developed. Therefore, there is an urgent need to find new alternative non-toxic antibacterial strategies able to deal with multidrug-resistant bacteria. Thus, the most common antimicrobial strategies usually include the utilization of antibiotics [4], metal ions and metal oxides [5], antimicrobial peptides (AMPs) [6], α-peptides [7], β-peptides [8], peptoids [9], and quaternary ammonium compounds (QACs) [10]. Antibiotics often show very low efficiency due to AR [11]. Metal ions and metal oxides have shown to be toxic for diverse types of mammalian cells [12]. QACs have also exhibited low efficiency due to drug resistance [13]. Peptides and peptoids are very promising but are still under industrial development [14]. However, the use of nanotechnology against multidrug-resistant pathogens has shown to be a very promising approach [15,16,17]. Furthermore, the antibacterial action of carbon nanomaterials is usually attributed to diverse hypotheses: membrane disruption, bacteria wrapping, electron transfer and induction of oxidative stress by reactive oxygen species. Some of these hyphotheses are based on physical mechanisms, resistances to which would be difficult for bacteria to develop [18,19]. Thus, carbon nanomaterials (CNMs) such as graphene oxide (GO) [20] and carbon nanofibers (CNFs) [21] have demonstrated to be a non-cytotoxic promising choice for a next-generation of antibacterial agents against clinically relevant Gram-positive pathogens [20,21]. Many studies have also confirmed the antibacterial activity of GO against Gram-negative bacterial strains [22,23,24] and, thus, it has been proposed as a new nanoweapon to combat multidrug-resistance bacteria [25] as a substitute to antibiotics [16]. However, the antibacterial activity and non-cytotoxicity of CNFs have recently been demonstrated under certain experimental conditions [21] and for GO, these properties are still open questions which demand further investigation because there are many controversial results. Thus, for example, GO dispersions have shown no antibacterial activity [26] and Ruiz et al. even reported that bacteria grew faster in the presence of GO because it stimulated the bacterial growth by acting as a surface to their attachment and proliferation [27]. Besides, many studies have shown that GO is toxic for some types of human cells [28,29]. 

From the CNMs family, GO is the most utilized nanomaterial [30] because it is environmentally-friendly and it is the easiest processing nanomaterial due to its abundant functional oxygen-containing groups, which render easier its dispersion in water. In addition, GO is now produced at a large scale and it of lower cost than other CNMs such as graphene [22]. In biomedicine, GO has shown excellent results in cancer treatment [31,32] and, in the field of antibacterial materials, silver nanoparticles (AgNPs) [33], graphene oxide-silver nanocomposites [34] and antimicrobial peptide-conjugated graphene oxide membranes [35] have shown to be powerful weapons against the multidrug-resistant bacteria methicillin-resistant *Staphylococcus aureus* (MRSA). However, compared with GO, carbon nanofibers (CNFs) have much more electric conductivity, which can be utilised to produce conductive composite materials [36], and a much lower cost [37]. Carbon nanofibers possess outstanding chemical, mechanical and electric properties, and present a quasi-one-dimensional morphology in the form of filaments [38,39]. Besides, we have recently reported that the addition of a minuscule amount of GO or CNFs into calcium alginate films can improve their water diffusion and compression properties significantly [37,40]. Other physical properties such as wettability, tensile strength and the thermal properties of hydrogels with different chemical natures have also exhibited a significant enhancement with the incorporation of CNMs [41,42,43,44].

On the other hand, light has shown to be a powerful tool to enhance bacterial killing [45]. Thus, the combination of 660 nm visible light together with AgNPs uniformly distributed into graphene oxide nanosheets wrapped with a thin layer of type I collagen have also shown strong antibacterial activity against *Escherichia coli* and *Staphylococcus aureus* [46]. However, the ability of bacteria to develop resistance mechanisms against Ag^+^ [47] and the high production cost of producing pure AgNPs combined with GO limit the potential use of these strategies.

Thus, according to our previous results obtained with GO/alginate [20] and CNFs [21], in this study, we expect that these carbon nanomaterial will be able to kill two clinically relevant Gram-positive multidrug-resistant pathogens: methicillin-resistant *Staphylococcus epidermidis* (MRSE) and methicillin-resistant *S. aureus* (MRSA), following the same successful procedure reported by other authors for GO against the Gram-negative *E. coli* bacterium [24]. Furthermore, since it has recently been reported that the antibacterial activity of GO can be enhanced with simulated sunlight against *E. coli* [48] and even though Gram-positive and Gram-negative cell walls differ much, we also hypothesize here that the antibacterial activity of GO and CNFs against MRSE and MRSA could also be enhanced under another type of illumination, light-emitting diode (LED), which has shown to possess great potential in the biomedical field in photo rejuvenation applications, treatment of several physical abnormalities and disorders, stress relief and dentistry [49,50]. Besides, in comparison with sunlight, LED light technology shows the great advantage of being able to produce continuous light without time and weather restrictions and can be easily combined with the nanotechnology of GO and CNFs in the development of new illuminated antibacterial biomedical materials and devices. 

MRSE is a multidrug-resistant bacterium spreading globally [51] and is currently one of the most important pathogens, especially among low-birth-weight premature infants [52]. *S. aureus* is a major human pathogen able to easily develop resistance to most antibiotics [53]. Furthermore, *S. epidermidis* and *S. aureus* currently constitute a leading cause of serious global health problems in diseases associated with medical instruments and catheters [54] due to their abundant presence on the skin of every human individual [55]. Therefore, since MRSE and MRSA are positioned between the most clinically relevant pathogens in the human skin, the cytotoxicity of CNFs and GO in contact with human keratinocyte HaCaT cells will also be analyzed in this study. 

## 2. Results and Discussion

**Figure 1:**
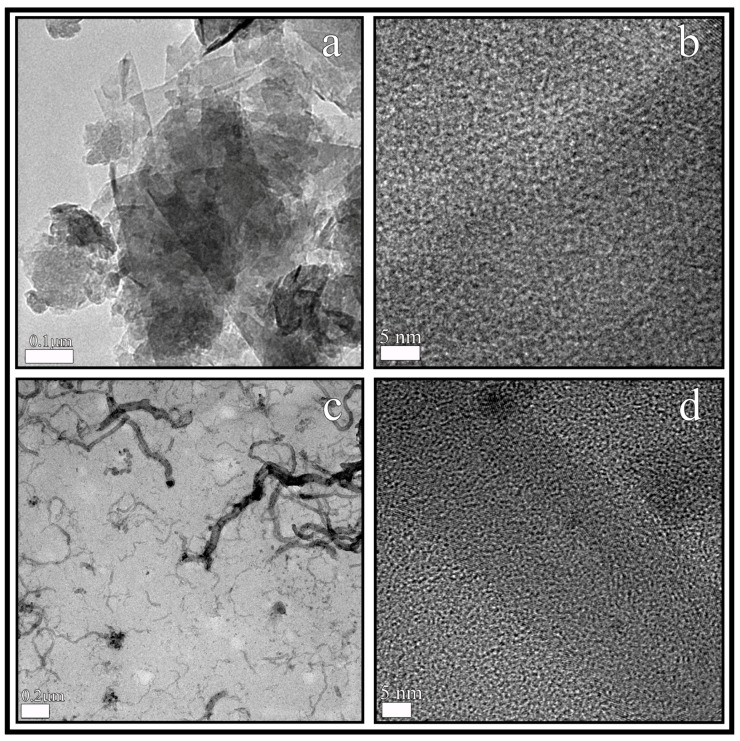
High-resolution transmission electron microscopy of graphene oxide nanosheets (**a**,**b**) and carbon nanofibers (**c**,**d**) at two different magnifications.

Using high-resolution transmission electron microscopy (HR-TEM), in this study, the GO and CNFs show a morphology of approximately 100–200 nm sheets (Figure 1a) and carbon nanofibers with irregular diameters of approximately 10–50 nm and lengths varying from some nm to several µm (Figure 1c) respectively.

The HR-TEM micrographs of the GO and CNFs at higher magnification both show black spots (Figure 1b,d), which were measured with the microscope’s software in order to confirm 3.42 Å of distance between the carbon atoms [56]. In addition, the EDS analysis showed that GO and CNFs are composed only of oxygen and carbon atoms. The carbon composition was 96.9 weight % (97.7 atomic %) for GO and 96.2 weight % (97.2 atomic %) for CNFs.

**Figure 2:**
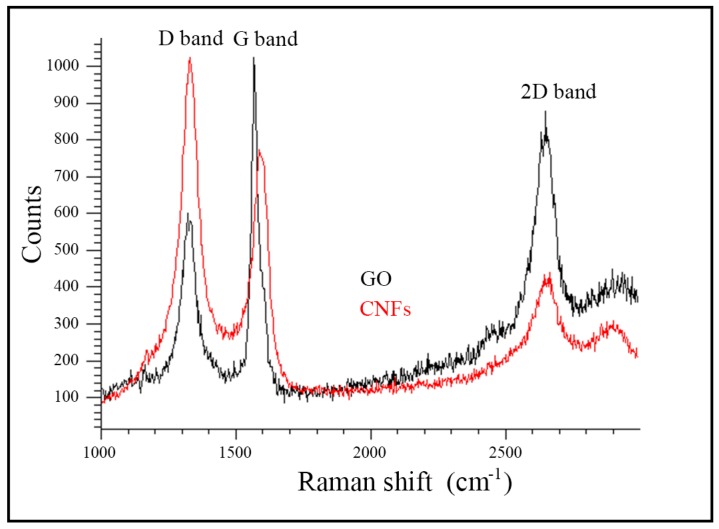
Raman spectrum of graphene oxide (GO) and carbon nanofibers (CNFs). The D, G and 2D bands are indicated.

Raman spectroscopy is commonly utilized to obtain the structural information of CNMs [57,58]. Thus, the Raman spectroscopy of the GO and CNFs used in this study showed a broad D band and a strong G band at approximately 1330 and 1580 cm^−1^ respectively as expected [57,58] (Figure 2). Structural defects, edge effects and dangling sp^2^ C bonds break symmetry in CNMs and produce a disordered band (the D band). The intensity ratio of the D and G bands (I_D_/I_G_) usually measures the defect/disordered carbon structure [59,60]. Thus, here, the I_D_/I_G_ ratio showed a value of 0.93 typical of GO [57] and 1.49 for CNFs confirming a higher degree of disorder present in the filamentous material, typical of irregular carbon structures [58]. Moreover, the 2D band at approximately 2660 cm^−1^ also indicates a significant degree of disorder [58] in both the nanomaterials supporting the different chemical structure of GO and CNFs with different oxygenated functional groups randomly distributed.

The antibacterial properties of GO and CNFs against the multidrug-resistant Gram-positive MRSE and MRSA bacteria were studied by dispersing the nanomaterials in isotonic saline solution at 80 µg/mL to directly interact with the bacterial cells for 1.5 and 3 h, as successfully performed previously with GO against Gram-negative *E. coli* [24] and CNFs against Gram-positive MRSE [21]. The results of these tests were compared with a negative control of MRSE and MRSA cells cultured without nanomaterials in isotonic saline solution and with a positive control of a well-known antibacterial agent (zinc). 

**Figure 3:**
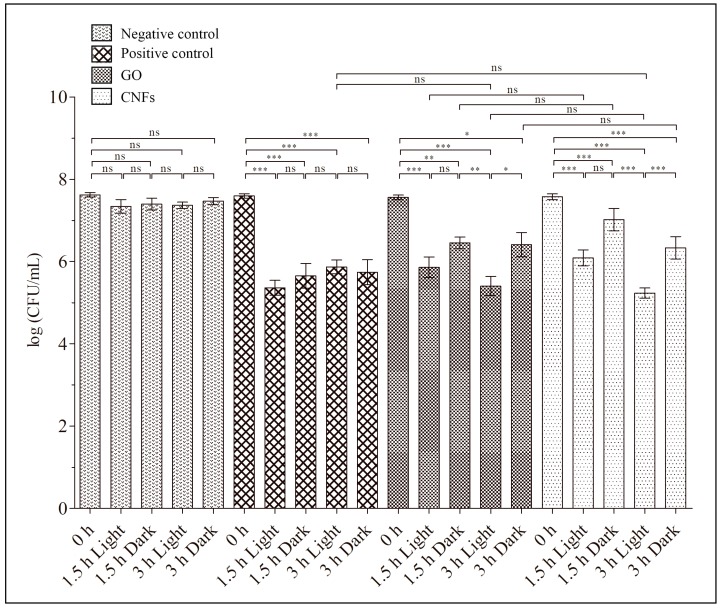
Methicillin-resistant *Staphylococcus epidermidis* (MRSE) cultured in isotonic saline solution (negative control), and in isotonic saline solution with 80 µg/mL of graphene oxide (GO), carbon nanofibers (CNFs) or zinc (positive control) at time zero and after 1.5 and 3 h of culture at 37 °C in the dark and under continuous LED irradiation. Error bars represent standard deviation. The ANOVA results are indicated in this plot. * *p* > 0.05; ** *p* > 0.01; *** *p* > 0.001; ns: not significant.

Figure 3 shows that MRSE bacteria lost viability in the presence of both GO and CNFs, which were evaluated by colony counting method. The effective antibacterial activity after 3 h was similar to that produced by zinc. Thus, the loss of viability shown in Figure 3 occurred as a decrease in colony forming units per mL (CFU/mL) from 0 to 3 h. The results, using Equation (1) to calculate for exposure time in the dark, were 87.33 ± 12.12% for GO, 95.68 ± 3.58% for CNFs and 96.74 ± 3.79% for zinc.

(1) $$LV\left(\%\right)=\frac{C_{0}-C}{C_{0}}\times 100$$

Furthermore, Figure 3 shows the significant enhancement of antibacterial activity when combining GO nanosheets or CNFs with LED irradiation after 3 hours. However, no statistically significant differences of antibacterial activity were observed between both CNMs in terms of a decrease in CFU/mL. Thus, the loss of viability of the MRSE cells, determined from the decrease in CFU/mL from time 0 to 3 h by applying Equation (1), was 98.5 ± 1.6% and 99.5 ± 0.6% for the GO and CNFs, respectively, under continuous LED light. 

The negative control samples were cultured in isotonic saline solution for 3 h. Thus, a slight decrease or no increase in bacterial viability of the negative controls was expected due to the lack of nutrients. No effect of LED irradiation was observed for the negative or positive controls. 

**Figure 4:**
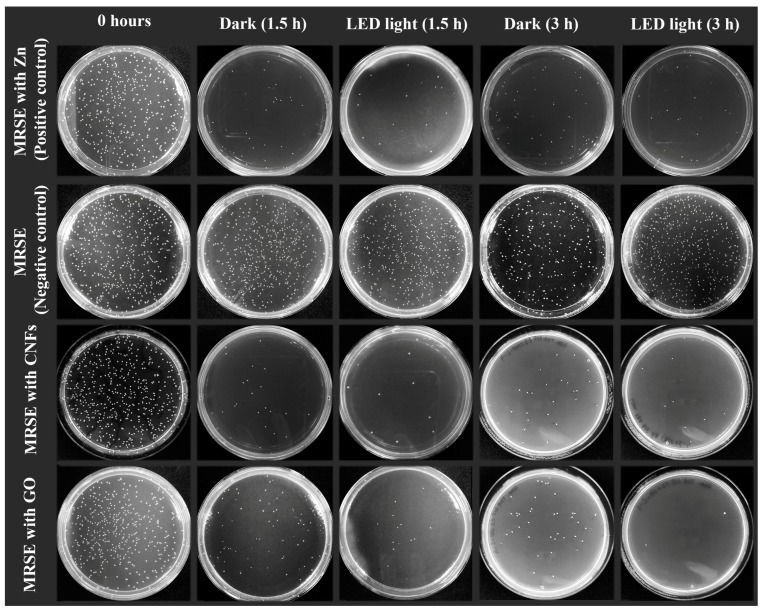
Representative plate photographs of the colony forming units (CFUs) for the antibacterial tests performed with methicillin-resistant *Staphylococcus epidermidis* (MRSE) in isotonic saline solution (negative control), and in isotonic saline solution with 80 µg/mL of graphene oxide (GO), carbon nanofibers (CNFs) or zinc (positive control) after 1.5 and 3 h of culture at 37 °C in the dark and under continuous LED irradiation (Dilution factor of 10^−4^).

Figure 4 shows representative plate photographs of the antibacterial tests for both nanomaterials and control cultures with MRSE at 0 h and after 1.5 and 3 h of culture at 37 °C. 

**Figure 5:**
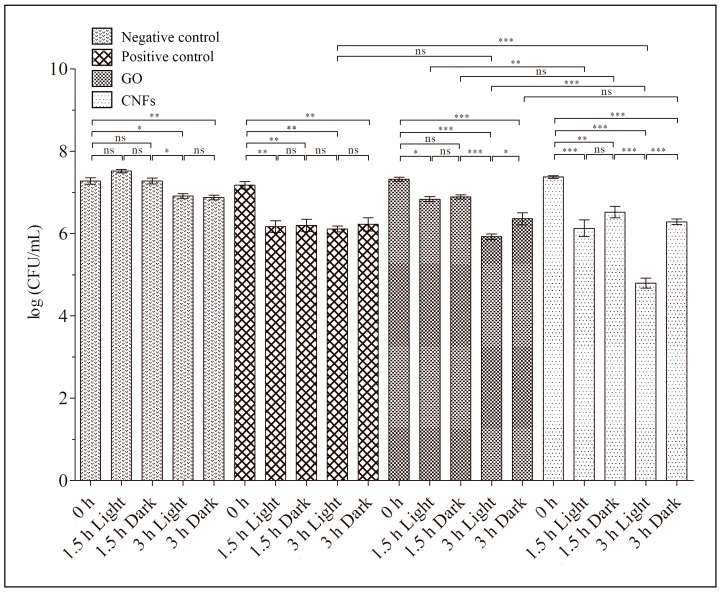
Methicillin-resistant *Staphylococcus aureus* (MRSA) cultured in isotonic saline solution (negative control), and in isotonic saline solution with 80 µg/mL of graphene oxide (GO), carbon nanofibers (CNFs) or zinc (positive control) at time zero and after 1.5 and 3 h of culture at 37 °C in the dark and under continuous LED irradiation. Error bars represent standard deviation. The ANOVA results are indicated in this plot. * *p* > 0.05; ** *p* > 0.01; *** *p* > 0.001; ns: not significant.

The loss of viability of the other Gram-positive multidrug-resistant strain, MRSA, in the presence of GO or CNFs, evaluated by the colony counting method, also exhibited effective antibacterial activity after 3 h in a similar way to that produced by zinc (Figure 5). Thus, the loss of viability, using Equation (1) to calculate for exposure time in the dark, was 87.05 ± 7.75% for GO, 91.22 ± 3.53% for CNFs and 87.35 ± 6.41% for zinc.

These results also show the significant enhancement of antibacterial activity when these CNMs were illuminated with a LED light lamp after 3 h. In addition, LED irradiation statistically showed more effect in combination with the filamentous CNFs than with GO nanosheets against MRSA in terms of a decrease in the logarithm of CFU per mL for both culture times (1.5 and 3 h). Thus, the loss of viability of the MRSA cells, calculated from the results shown in Figure 5 as the decrease in CFU/mL from time 0 to 1.5 and 3 h, by applying Equation (1), showed that MRSA cultivated in isotonic saline solution with GO or CNFs in combination with continuous LED illumination was 66.1 ± 8.1% and 87.1 ± 4.5%, respectively for 1.5 h and 95.8 ± 1.4% and 99.7 ± 0.2% respectively for 3 h. This slight increase in antibacterial activity could be attributed to the fact that filamentous carbon nanomaterials possess a larger surface area [61] exposed to the LED irradiation than graphene oxide nanosheets. However, the greater effect of LED light on CNFs than on GO was not observed with the other multidrug-resistant strain (Figure 3). 

The negative control samples were cultured in isotonic saline solution for 3 h. Thus, a slight decrease or no growth of bacterial viability of the negative controls was expected due to the lack of nutrients. No effect of LED irradiation was observed for the negative or positive controls. 

All these results are in good agreement with those previously published for GO against Gram-negative *E. coli* for the same experimental conditions (concentration and time exposures) [24]. 

**Figure 6:**
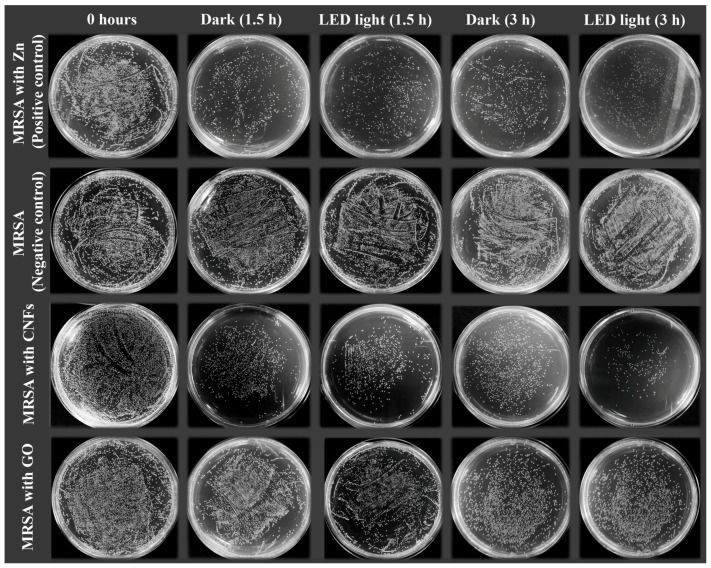
Representative plate photographs of the colony forming units (CFUs) for the antibacterial tests performed with methicillin-resistant *Staphylococcus aureus* (MRSA) in isotonic saline solution (negative control), and in isotonic saline solution with 80 µg/mL of graphene oxide (GO), carbon nanofibers (CNFs) or zinc (positive control) after 1.5 and 3 h of culture at 37 °C in the dark and under continuous LED irradiation (Dilution factor of 10^−2^).

Figure 6 shows representative plate photographs of the antibacterial tests for the CNMs dispersed in isotonic saline solution at 80 µg/mL in direct contact with the MRSA cells after 1.5 and 3 h of culture at 37 °C in the dark and under continuous LED illumination.

**Figure 7:**
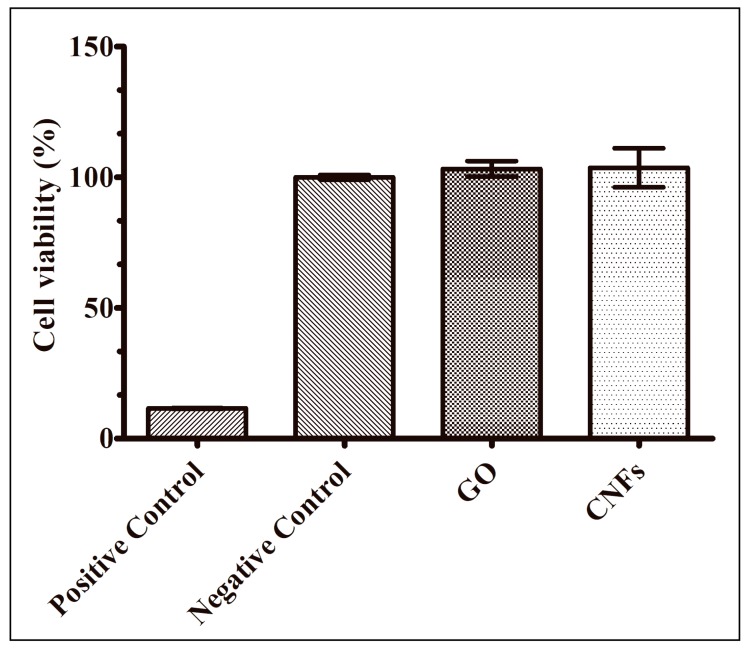
Cytotoxicity results (MTT assay) for graphene oxide (GO) and carbon nanofibers (CNFs) dispersed in isotonic saline solutions, isotonic saline solution with zinc (positive control) and isotonic saline solution (negative control) in the presence of human keratinocyte HaCaT cells at 37 °C for 3 h. No statistically significant (*p* < 0.01) differences of cell viability were found between the negative control and GO or CNF sample cultures.

Finally, the methylthiazolydiphenyl-tetrazolium bromide (MTT) assays of the CNMs showed no statistically significant cytotoxicity in the presence of human keratinocyte HaCaT cells for 3 h of culture at 37 °C (Figure 7).

These results confirm our hypotheses that the antibacterial activity of GO and CNFs can be enhanced under continuous LED light against the Gram-positive multidrug-resistant MRSE and MRSA pathogens. LED irradiation must accelerate the electron transfer from antioxidant biomolecules to the CNMs, which simultaneously destroys bacterial antioxidant systems and produces the reduction of the CNMs as reported upon exposure to simulated sunlight on GO [48]. The successful results obtained against *E. coli* with simulated sunlight, and, here against MRSE and MRSA with LED irradiation, broaden the specificity of this antibacterial strategy. 

The relatively long treatment period (1.5 or 3 h) of these combined nanotechnologies could limit their utility for surface or device disinfection. However, these results open up new exciting opportunities for potential clinical application where LED light technology can be utilized in combination with the nanotechnology of GO or CNFs as novel antibacterial nanoweapons for the prevention and treatment of Gram-positive multidrug-resistant infections. 

## 3. Materials and Methods

### 3.1. Carbon Nanomaterials

Graphene oxide powder (GO, 15–20 sheets, 4–10% edge-oxidized, Sigma-Aldrich, St. Louis, MO, USA) and carbon nanofibers (CNFs, Graphenano, Yecla, Spain) were used as received. 

### 3.2. Electron Microscopy

GO nanosheets and carbon nanofibers were observed in a JEM 2100F 200 kV high-resolution transmission electron microscope (HR-TEM, JEOL, Tokyo, Japan). This equipment is provided with energy-disperse X-ray spectroscopy (EDS) to determine elemental compositions at 20 kV. The carbon nanomaterials were ultrasonically dispersed in dichloromethane for 10 min and after that, they were dried at ambient temperature before HR-TEM observation. 

### 3.3. Raman Spectroscopy

Raman spectroscopy was performed from 1000 to 3000 cm^−1^ in a Renishaw inVia confocal micro-Raman spectrometer using an argon ion laser at 633 nm and ×20 lens at 600 L·mm^−1^ grating. The GO or CNFs powder was deposited onto a glass disk for direct observation.

### 3.4. Antibacterial Test

Graphene oxide exhibited significant antibacterial action against Gram-negative *E. coli* bacterium, at 80 µg/mL, dispersed in isotonic saline solution (to remove chemical interactions with medium compounds) and 3 h of direct contact [24]. Therefore, according to the procedure described in that work, sterile samples of GO or CNFs dispersed in isotonic saline solution (NaCl 0.9% *w*/*v*), from FisioVet (B. Braun VetCare SA, Barcelona, Spain) at the same concentration (80 µg/mL), and autoclaved at 121 °C for 15 min, were prepared by sonication for two hours right before being used for the bacterial cultures. The methicillin-resistant *Staphylococcus epidermidis*, RP62A [62], and the methicillin-resistant *Staphylococcus aureus*, COL [63], Gram-positive bacterial strains were utilized for the antibacterial tests. Thus, bacterial cultures were used in TSB in a shaking incubator (140 rpm and 37 °C) overnight. Cell samples of 10^6^ to 10^7^ CFU/mL were prepared by dilution of these cultures and harvestation in the mid-exponential growth phase. Bacterial concentrations were determined with a Nanocolor UV0245 UV/VIS spectrophotometer (Macherey-Nagel, Düren, Germany) at 540 nm. In order to pellet cells, the cultures were centrifuged at 6000 rpm (3952× *g*) for 10 min. After centrifugation, cells were washed with isotonic saline solution three times to ensure complete elimination of any type of residual molecules such as growth medium compounds. The bacterial cells were then resuspended in fresh sterile GO dispersion to be incubated at 37 °C under 250 rpm shaking speed for 1.5 and 3 h. The loss of viability of the microorganisms was evaluated by the colony counting method [64] after those two selected times. Thus, 100 μL of each cell dilution was spread onto TSA plates and left to grow overnight at 37 °C. Colonies were counted and compared with those grown on negative control plates, which were obtained from the control bacterial cultures without GO or CNFs. Furthermore, a positive control culture was used with a well-known antibacterial agent (zinc [65]). In this positive control, zinc chloride (≥97.0%, Sigma-Aldrich) was dissolved in isotonic saline solution to a concentration of 80 µg/mL. The microorganism cultures were placed in 15 mL transparent tubes placed, in the vertical position in the dark (covered with aluminum foil) and under continuous light using a 50W 4000 lumens LED lamp (F-Bright Led, Bizkaia, Loiu, Spain) with Daylight White 6000K Color and 285 × 54 × 240 mm dimensions, located at a distance of 34.7 cm from the culture on the top of a transparent Certomat IS orbital shaking incubator (Sartorius Stedim Biotech, Göttingen, Germany) at 37 °C for 3 h. The amount of light at this distance was 10710 Lux, which was measured with a DVM1300 Velleman light meter (Velleman, Inc., Fort Worth, TX, USA). 

Loss of viability (*LV*) can be determined as the decrease in CFU/mL from time zero (*C_0_*) to 1.5 or 3 h of culture (*C*) divided by the initial concentration (*C_0_*) as expressed in Equation (1) [24].

These antibacterial tests were performed in triplicate in three different days (*n* = 9) to provide reproducibility.

### 3.5. MTT Cytotoxicity Assay for GO and CNFs

Human keratinocyte HaCaT cells were provided by the Medical Research Institute Hospital La Fe (Valencia, Spain) for the cytotoxicity assays. The cell viability of these human cells was evaluated in the presence of GO or CNFs by the methylthiazolyldiphenyl-tetrazolium bromide (MTT) test. Cell culture was performed in a 96-well plate at a density of 5 × 10^5^ cells/well. After 24 hours of incubation, the culture medium of each well was replaced with 100 µL dispersions of GO or CNFs into isotonic saline solution at the same concentration that was used for the antibacterial tests (80 µg/mL). The dispersion of the nanomaterials was produced by 2 h sonication and always used immediately. 100 µL of pure isotonic saline solution (without GO or CNFs) and 100 µL of isotonic saline solution with a high concentration of zinc (1000 µM), that is over the toxicity level for mammalian cells [66], were also used to replace the culture medium as negative and positive controls, respectively. After 3 h of incubation (maximum time exposure in the antibacterial assays), the cells were incubated with 5 mg/mL MTT in each well for 4 h. Finally, the formazan was dissolved in 100 µL of dimethyl sulfoxide at 24 ± 1 °C and absorbance readings were carried out at 550 nm on a microplate reader (Varioskan, Thermo Fisher, Waltham, MA, USA). 

### 3.6. Statistical Analysis

The results obtained in this study were statistically analyzed by ANOVA followed by multiple Tukey’s post-hoc analysis using the GraphPad Prism 6 software (San Diego, CA, USA) at significance level of at a least *p* < 0.05. 

## 4. Conclusions

This study has shown that graphene oxide nanosheets and carbon nanofibers are valuable antibacterial carbon nanomaterials able to kill two clinically relevant Gram-positive multidrug-resistant strains: methicillin-resistant *Staphylococcus epidermidis* and methicillin-resistant *Staphylococcus aureus*. Furthermore, LED light technology was shown to be able to significantly enhance the antibacterial properties of graphene oxide nanosheets and carbon nanofibers against these life-threatening pathogens. Therefore, these carbon nanomaterials in combination with LED irradiation for 3 h periods had an efficient antibacterial effect against MRSE and MRSA pathogens. The implementation of these combined technologies opens up a wide range of research opportunities in many important fields within the biomedical and bioengineering sector.

## Abbreviations

LED	Light-emitting diode
GO	Graphene oxide
CNFs	Carbon nanofibers
CNMs	Carbon nanomaterials
MRSA	Methicillin-resistant *Staphylococcus aureus*
MRSE	Methicillin-resistant *Staphylococcus epidermidis*
AR	Antibiotic resistance
AMPs	Antimicrobial peptides
HR-TEM	High-resolution transmission electron microscopy
EDS	Energy-disperse X-ray spectroscopy
CFUs	Colony-forming units
QAC	Quaternary ammonium compounds
AgNPs	Silver nanoparticles
LV	Loss of viability
